# Current measurements in the intermittent-contact mode of atomic force microscopy using the Fourier method: a feasibility analysis

**DOI:** 10.3762/bjnano.11.37

**Published:** 2020-03-13

**Authors:** Berkin Uluutku, Santiago D Solares

**Affiliations:** 1 The George Washington University, Department of Mechanical and Aerospace Engineering, 800 22nd St. NW, Suite 3000, Washington, DC 20052, USA

**Keywords:** atomic force microscopy (AFM), conductivity, current, intermittent contact, Fourier analysis, tapping-mode AFM

## Abstract

Atomic force microscopy (AFM) is an important tool for measuring a variety of nanoscale surface properties, such as topography, viscoelasticity, electrical potential and conductivity. Some of these properties are measured using contact methods (static contact or intermittent contact), while others are measured using noncontact methods. Some properties can be measured using different approaches. Conductivity, in particular, is mapped using the contact-mode method. However, this modality can be destructive to delicate samples, since it involves continuously dragging the cantilever tip on the surface during the raster scan, while a constant tip–sample force is applied. In this paper we discuss a possible approach to develop an intermittent-contact conductive AFM mode based on Fourier analysis, whereby the measured current response consists of higher harmonics of the cantilever oscillation frequency. Such an approach may enable the characterization of soft samples with less damage than contact-mode imaging. To explore its feasibility, we derive the analytical form of the tip–sample current that would be obtained for attractive (noncontact) and repulsive (intermittent-contact) dynamic AFM characterization, and compare it with results obtained from numerical simulations. Although significant instrumentation challenges are anticipated, the modelling results are promising and suggest that Fourier-based higher-harmonics current measurement may enable the development of a reliable intermittent-contact conductive AFM method.

## Introduction

Conductive atomic force microscopy (C-AFM), a contact-mode technique, has been extensively utilized to investigate local electrical properties of nanoscale systems, such as organic solar cells [1–7], semiconductors [8–10], and metals [11–13]. C-AFM has been used to characterize local charge transport characteristics [4,6] and to obtain detailed information about local charge mobility [5,7]. However, contact-mode AFM techniques, where the probe continuously interacts with the surface in a repulsive manner, can be destructive to soft samples [14–16]. In fact, C-AFM has been deliberately used as an imprinting tool in the past [17–18]. For the cases where the sample is rather delicate, intermittent-contact mode (ICM) imaging, where the tip and the sample interact briefly at the bottom of each cantilever oscillation, can be a less destructive technique [16,19–20], and this could be advantageous also for performing current measurements on such samples. Additionally, scanning tunnelling microscopy (STM) applications may also benefit from current measurements during which the tip oscillates above the surface, although in the noncontact regime. Specifically, STM measurements are modulated based on the observed tunnelling current, which has an exponential dependence on the tip–sample distance [21]. Therefore, any unexpected contact with the surface may lead to a current spike and severely perturb the controller for a period of time, during which the tip apex structure could be damaged further due to additional tip–sample impacts. However, if a noncontact oscillatory current measurement mode is used, where the control variable is not the instantaneous value of the current, these unexpected tip–sample impacts may be more benign and may not perturb the measurement as drastically as in traditional STM approaches.

Intermittent-contact current measurement within AFM has already been discussed in the literature. A notable example is the work by Fein et al. where injected voltage pulses were investigated using a custom-made, low-frequency, high-stiffness cantilever [22]. Another example is the work of Vecchiola et al. where a “pulsed force” microscopy approach was implemented, rather than traditional ICM-AFM [23]. Although the end result was intermittent-contact characterization, due to the nature of the force pulses the probe jumped from contact point to contact point rather than exhibiting a constant, nearly resonant intermittent-contact oscillation (the oscillation frequencies used were much smaller than the resonance frequency of the cantilever).

In this paper, we propose the use of Fourier analysis to implement ICM current measurements. Fourier analysis is commonly used in ICM-AFM experiments due to the periodic nature of the cantilever excitation and response. For example, in amplitude-modulation AFM (AM-AFM), the most common ICM-AFM method, a lock-in amplifier is used to track the cantilever response near the fundamental frequency [20]. Similarly, bimodal AFM, which involves the excitation of the cantilever at two frequencies, also uses lock-in amplifiers or phase-locked loops to control or observe each frequency response [24–25]. More elaborate Fourier analysis techniques have also been implemented [26], such as in the work of Stark et al. where time-resolved transient forces between the AFM probe and the sample were obtained from the experimental data [27]. This approach was enhanced by Sahin et al. through a “torsional harmonic cantilever” [28], which combined flexural and torsional oscillations in a way that reduced cross-contamination of the signals used to reconstruct the tip–sample force. We have also reported numerical simulations of this method, providing analysis software that enables estimations of the accuracy of the method under different conditions [29]. Fourier analysis has also been implemented for AFM force reconstructions within the so-called intermodulation AFM method, developed by Haviland and co-workers, where the cantilever is typically excited simultaneously at two different frequencies, while various intermodulation products are recorded with a collection of lock-in amplifiers [30–31]. More recently, Borgani et al. used Fourier analysis to investigate non-linear conductance in C-AFM measurements, acquiring current–voltage responses at every scan point without sacrificing scanning speed [32].

In order to explore the various phenomena involved in dynamic current measurements, this manuscript discusses three different cases: (i) a noncontact dynamic current measurement where the cantilever follows an ideal sinusoidal trajectory, (ii) a similar case, but considering a more realistic trajectory where the tip oscillation is perturbed by the presence of the sample, and (iii) an intermittent-contact case where a Hertzian contact interaction is established with the sample and interrupted again during each cantilever oscillation. In the Results section, these three cases are simulated and the results are compared with the equations derived later in the current section. Practical and instrumentation challenges for the proposed methods in the context of real SPM experiments are summarised in the Discussion section, such as data acquisition difficulties when multiple weak signals at high frequencies are measured. Possible solutions are also discussed in some cases, although some of these challenges are significant and have not yet been overcome. In the remainder of this section we will derive Fourier space expressions for the measured current for each case analysed.

## Case 1: Dynamic noncontact current measurement with ideal sinusoidal tip trajectory

**Figure 1:**
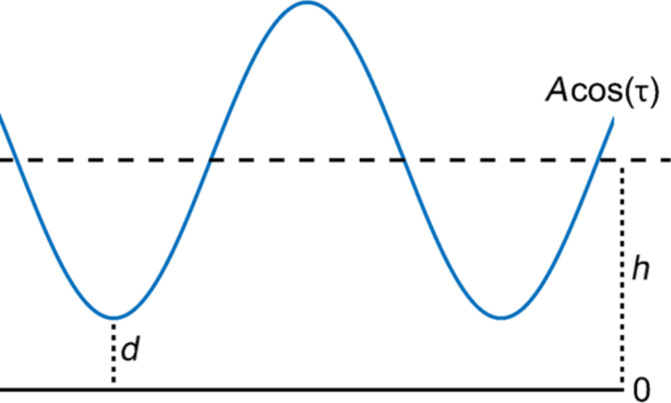
Illustration of a tip trajectory with a perfect sinusoidal shape in the noncontact dynamic AFM mode. The blue line represents the tip motion about the equilibrium position of the cantilever, while the solid black line represents the surface position, fixed at reference point zero. *h* is the cantilever rest position and *d* is the instantaneous tip–sample distance.

Consider an AFM tip oscillating over a surface with a perfect cosine trajectory, without impacting the surface (Figure 1). In this case the distance between the AFM tip and the surface can be written as

[1] d=h+Acos(2π​fτ),

where *d* is the instantaneous tip–sample distance, *h* is the equilibrium tip position, *A* is the oscillation amplitude, *f* is the oscillation frequency and τ is time. Since there is no tip–surface contact, we consider a tunnelling current between the tip and the surface, which we approximate with an exponential function of the distance [21],

[2] $$J=\sigma {\text{e}}^{-Kd},$$

where σ is a linear scaling parameter and *K* provides the rate of exponential decay.

Inserting Equation 1 into Equation 2 we obtain an expression for the tunnelling current with respect to time:

[3] $$\begin{array}{c} J=\sigma {\text{e}}^{-K\left[h+A\cos\left(\tau \right)\right]}\\ =\sigma {\text{e}}^{-Kh}{\text{e}}^{-KA\cos\left(2\pi \text{​}f\tau \right)}\\ =\sigma {\text{e}}^{-Kh}{\text{e}}^{KA\cos\left(2\pi \text{​}f\tau +\pi \right)}. \end{array}$$

We expect to have maximum tunnelling current at the bottom of the oscillation, where the cosine is at its minimum (this is where the tip and sample are closest). Likewise, the lowest value of the current occurs when the cosine reaches its maximum value. Since the oscillation phase reference is arbitrary, it is convenient to replace the time variable as follows:

[4] 2π​fτ+π=2π​f​t,

[5] $$J=\sigma {\text{e}}^{-Kh}{\text{e}}^{KA\cos\left(2\pi \text{​}f\text{​}t\right)}.$$

Note that the left-hand side of Equation 4 depends on the variable τ, whereas the right-hand side depends on *t*. Expressions such as the right-hand side of Equation 5 can be expanded with a Fourier cosine series using the modified Bessel functions of the first kind (of different orders) as the Fourier coefficients [33]:

[6] $${\text{e}}^{z\cos\left(\theta \right)}=I_{0}\left(z\right)+2\sum_{k=1}^{\infty }I_{k}\left(z\right)\cos\left(\theta \right).$$

We apply this expansion to our current equation and obtain:

[7] $$J=\sigma {\text{e}}^{-Kh}\left[I_{0}\left(AK\right)+2\sum_{k=1}^{\infty }I_{k}\left(AK\right)\cos\left(2\pi \text{​}f\text{​}k\text{​}t\right)\right].$$

In order to be able to more intuitively analyse the result, we take the Fourier transform of the series in Equation 7:

[8]
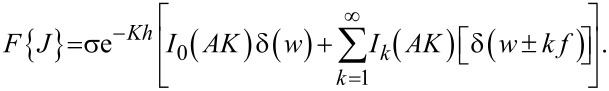


We will return to this expression in order to analyse and visualize it in the Results section.

## Case 2: Dynamic noncontact current measurement with realistic tip trajectory

A real AFM tip trajectory exhibits perturbations due to the tip–sample forces, which have been treated analytically by Dürig [34–36] and investigated further by several other researchers [37–39]. The perturbed tip trajectory can be expressed as a Fourier cosine series:

[9] $$\psi =\sum_{n=1}^{\infty }a_{n}\cos\left(2\pi n\text{​}f\text{​}t\right).$$

Here the cantilever response consists of the principal frequency oscillation plus its higher harmonics, with the *a_n_* values representing the amplitudes of those harmonics. For instance, *a*_1_ corresponds to the fundamental frequency of the cantilever response, which is typically tracked using a lock-in amplifier and modulated during a standard dynamic AFM experiment. As outlined in the work of Hembacher and co-workers [37], the *a_n_* values correspond to higher harmonics of the cantilever oscillation, as indicated in Equation 10, where the tip–sample interaction force exhibits short range compared to the full cantilever oscillation:

[10]
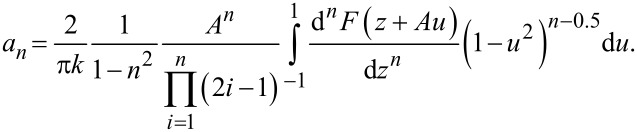


The *a_n_* values decrease rapidly with increasing *n* in conventional dynamic mode applications [note that in Equation 10 the *a_n_* values are proportional to the inverse of (1 − *n*^2^)]. The analysis of the cantilever trajectory and its higher harmonic responses are discussed in detail in [34–39]. The higher harmonics (i.e., *a*_2_, *a*_3_*,*…) have also been measured in dynamic AFM experiments [40–41].

For our cantilever trajectory we will use Equation 9, since a tip–sample force perturbation is present. Since we are still considering a noncontact case, we will use the tunnelling current model from Equation 3. The tip–sample distance is:

[11] $$d=h+\psi =h+\sum_{n=1}^{\infty }a_{n}\cos\left(2\pi n\text{​}f\text{​}t\right).$$

We treat this expression in a similar manner as for the previously analysed ideal case and obtain the following current expression in the time domain:

[12] $$\begin{array}{c} J=\sigma {\text{e}}^{K\psi }{\text{e}}^{-Kh}\\ =\sigma {\text{e}}^{-Kh}\prod_{n=1}^{\infty }\left[{\text{e}}^{Ka_{n}\cos\left(2\pi \text{​}f\text{​}nt\right)}\right]\\ =\sigma {\text{e}}^{-Kh}\prod_{n=1}^{\infty }\left[I_{0}\left(a_{n}K\right)+2\sum_{k=1}^{\infty }I_{k}\left(Ka_{n}\right)\cos\left(2\pi \text{​}f\text{​}nt\right)\right]. \end{array}$$

We can again easily apply the Fourier transform to find the frequency-domain representation of the tunnelling current. In this case, however, although it is trivial to obtain the Fourier transform of the Fourier series, we have an infinite number of different Fourier series multiplied with one another. These multiplications in the time domain correspond to convolutions in the frequency domain:

[13]
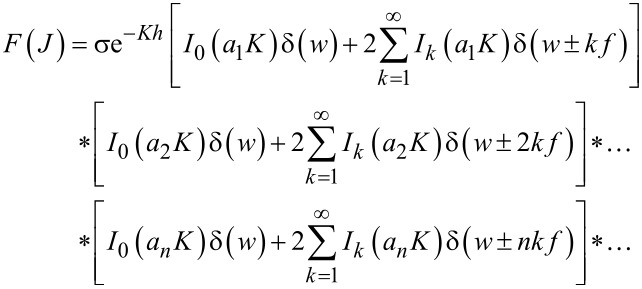


This infinite number of convolutions between infinite series may look intimidating at first glance. However, we note that the infinite series in Equation 13 consist of delta functions at different frequencies, multiplied by their respective coefficients. Convolution of a given function with a delta function yields only a time shift in the convolved function, without changing the original shape of the function. For instance,


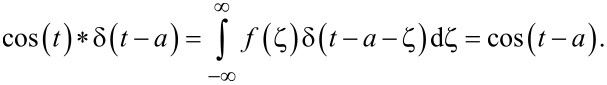


**Figure 2:**
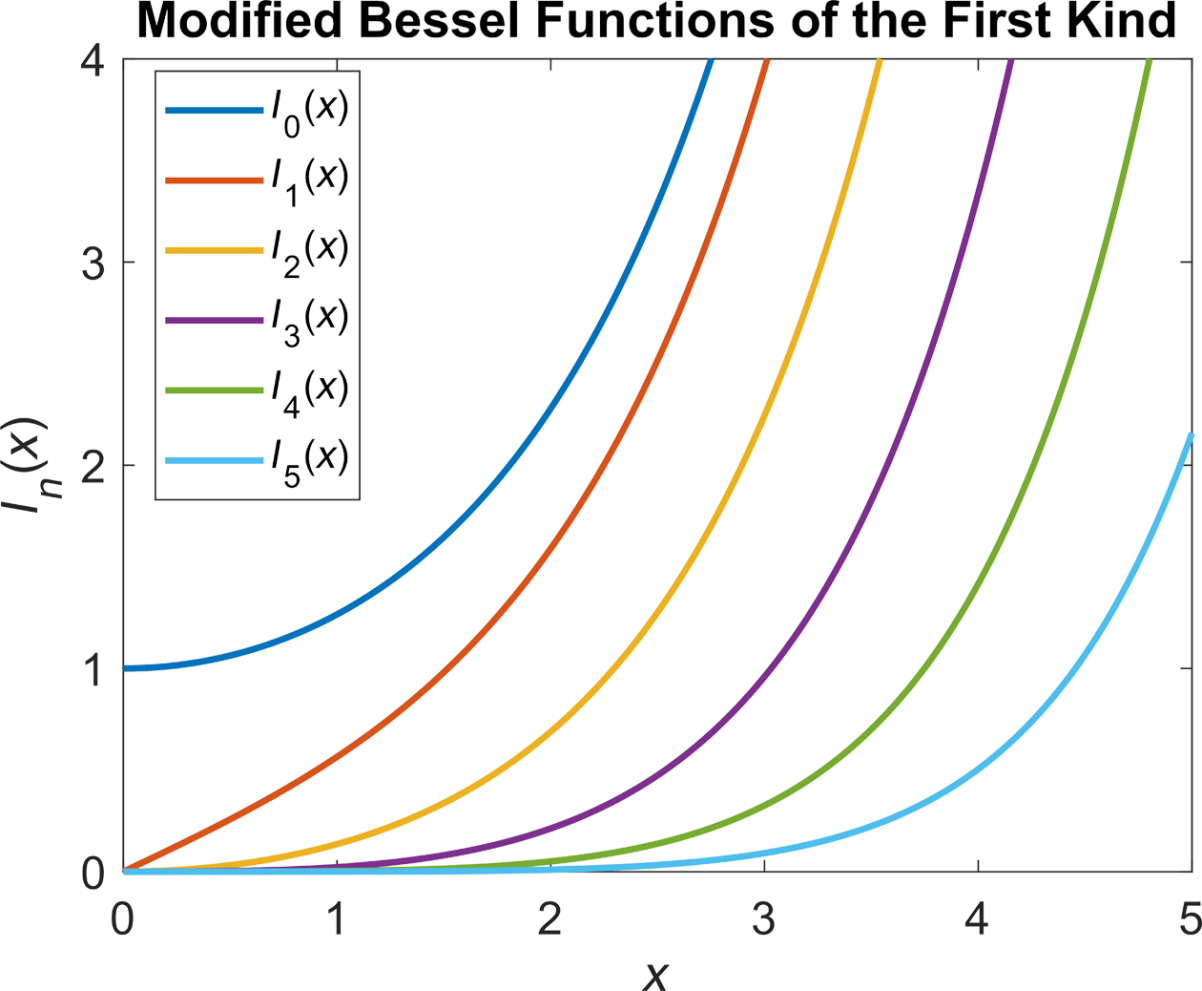
Modified Bessel functions of the first kind of different orders. While the zeroth-order function approaches unity at the origin, higher-order functions approach zero quite steeply. Higher-order functions converge to zero more quickly than their lower-order counterparts as the origin is approached from the right.

Furthermore, we know that the coefficients of the aforementioned delta functions correspond to modified Bessel functions of the first kind, which increase in order with every consecutive term in the infinite series. The zeroth-order modified Bessel function of the first kind approaches unity when its argument approaches zero. Higher-order modified Bessel functions of the first kind approach zero when their argument approaches zero (see Figure 2). Combining this knowledge with the knowledge of rapidly decreasing values of *a_n_*, we can conclude that the higher harmonics of the current will approach a delta function with a coefficient equal to unity at 0 Hz, which has no effect on convolution operations:

[14]
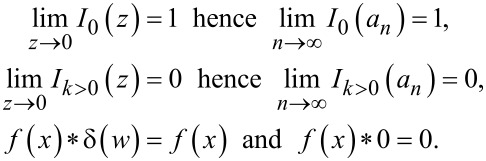


Thus, we can conclude that only the first few harmonics will contribute significantly. Intuitively, it is also expected that the higher harmonics should not contribute significantly to the final convolution based on their *a_n_* values approaching zero with increasing *n*.

## Case 3: Dynamic intermittent-contact current measurement with realistic tip trajectory

**Figure 3:**
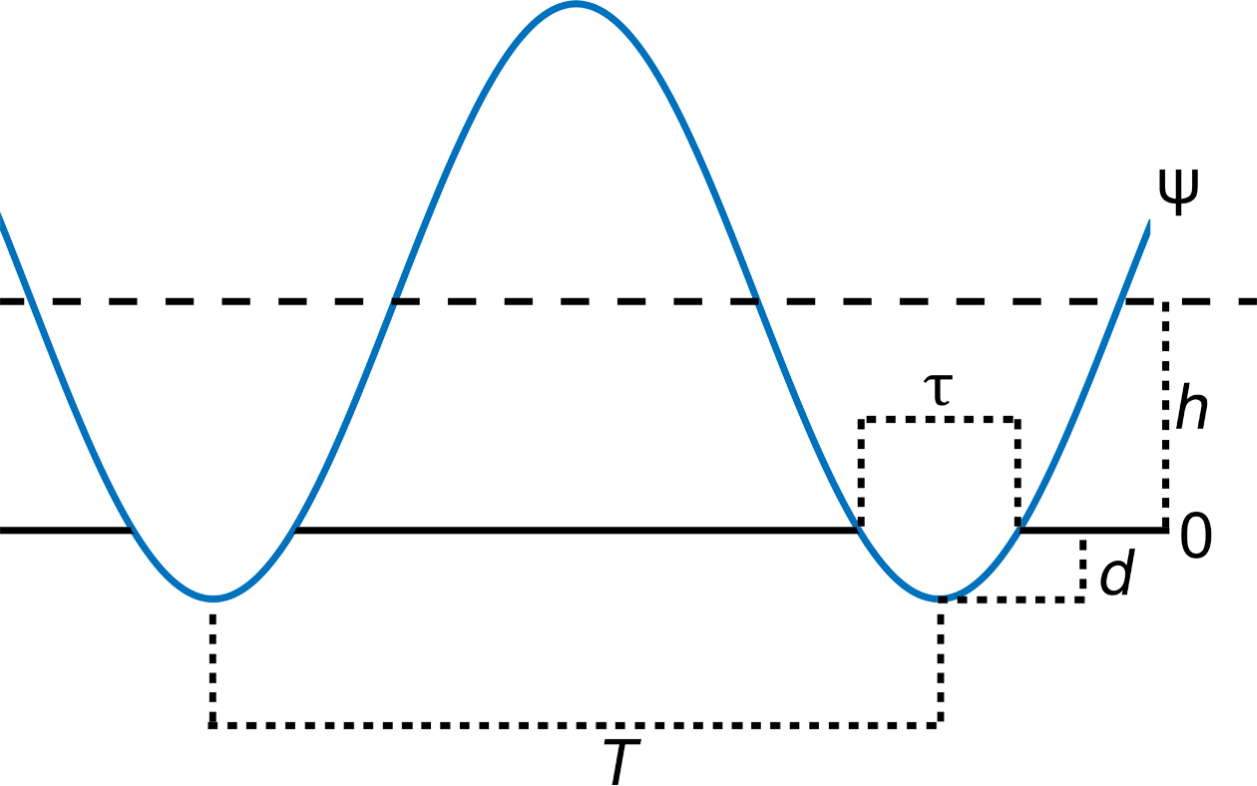
Illustration of the intermittent-contact interaction case. The blue line represents ψ, the trajectory of the tip about the equilibrium position of the cantilever, while the solid black line represents the surface position, fixed at reference point zero. *h* is the cantilever rest position, *T* is the fundamental period of the tip trajectory, *d* is the indentation, and 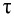 is the contact time.

Here we consider a tip trajectory such that intermittent tip–sample repulsive contact occurs, for which a much larger current is expected from the conduction (contact) sections of the trajectory than from the tunnelling (noncontact) sections of the trajectory. During the conduction phase, we will treat the current as being proportional to the contact area, as a first approximation, as in an Ohmic contact, and will neglect the small tunnelling current for simplicity. Using the Hertzian contact model [42] for the repulsive interaction and considering surface indentation, we can write the current as:

[15]
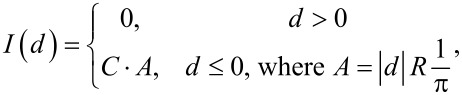


where *d* is the tip–sample distance, *C* is a conduction proportionality constant, and *R* is the AFM tip radius. The overall setting we have described is represented in Figure 3.

**Figure 4:**
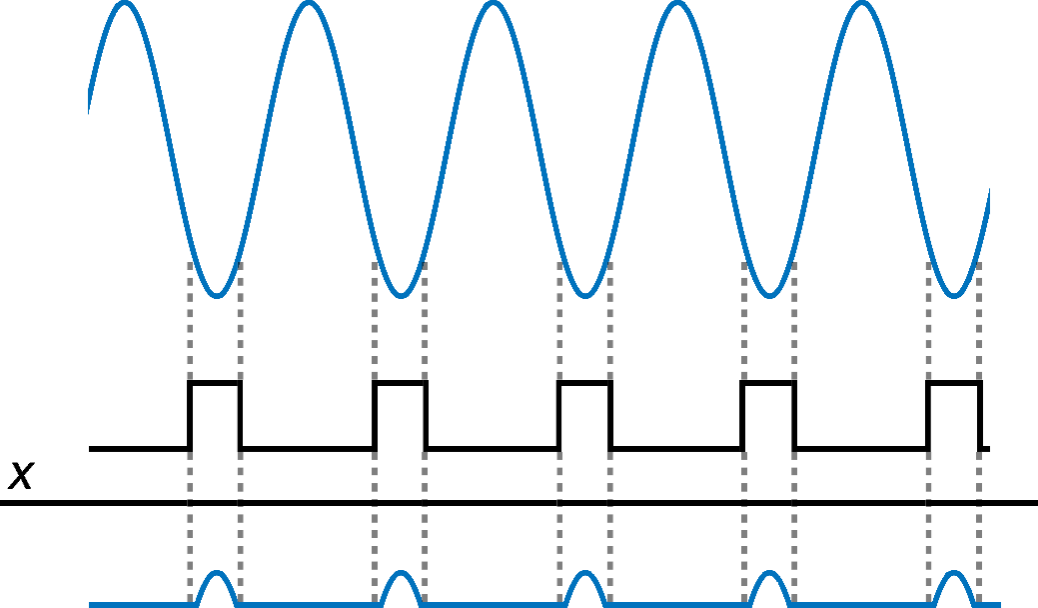
Illustration of the derivation of the indentation. The upper blue line represents the tip–sample distance. Multiplying that function with a square pulse function (black line) with duty cycle equal to the contact time, and changing sign, yields the indentation, represented by the lower blue line.

Although frequency-domain analysis has provided useful insight and mathematical convenience for the previous two cases considered, it is more challenging to perform here due to the piecewise expression for the current. We can address this issue using the square wave function (sq) to represent the indentation, *In*, as follows:

[16] In=−d⋅sq(τ,T)=−(h+ψ)⋅sq(τ,T),

where ψ is the tip trajectory, and sq(τ,*T*) is the square wave function with period *T* and duty cycle τ, ranging from zero to unity and with a duty cycle centred around zero time [43]. It is clear that τ, the duty cycle of the square wave function, corresponds to the effective interaction time between the tip and the sample. Upon introduction of sq(τ,*T*), which has a well-defined Fourier transform, our indentation will automatically be zero whenever there is a positive tip–sample distance (see Figure 4). We can now define the current as

[17] $$I\left(In\right)=In\cdot C\cdot R\cdot \frac{1}{\pi }$$

and take its Fourier transform:

[18]
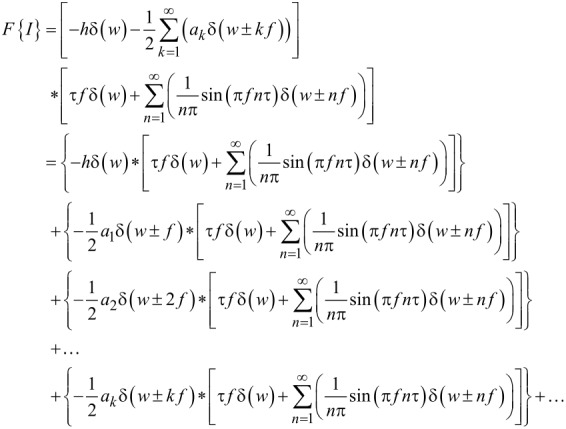


In the above equation, two terms (in brackets) are being convoluted. The first term corresponds to the tip trajectory and the second term comes from the introduction of the square pulse. One can see that the contribution coming from the square pulse function has a sinc-shaped envelope on the coefficients of the delta functions, (1/*n*π)·sin(π*fn*τ). Examination of the term coming from the trajectory reveals that it can be separated into two components: a zero frequency (DC) contribution and a harmonics contribution. The DC contribution corresponds to the term containing δ(*w*) and the harmonics contribution corresponds to the terms containing δ(*w* ± *nf*) inside the summation. Due to the additive property of the convolution, this operation can be performed one component at a time. This means that individual components of the motion can be convolved with the term originating from the square wave function and summed up afterwards. The zero-frequency contribution will yield a comb of delta functions with a sinc envelope on their coefficients, coming from the square wave function, while each of the delta functions deriving from the harmonics of the motion will lead to a shifted comb of delta functions with rescaled coefficients governed by a sinc envelope. This is demonstrated in Equation 19 and Equation 20. Equation 19 shows the expansion of the term coming from the DC contribution in the trajectory. As the equation shows, convolving the contribution of the rectangular pulse with the 0 Hz part of the trajectory only rescales the former. Equation 20 illustrates the convolution of the square pulse contribution with the first harmonic coming from the trajectory. This operation both rescales and shifts the square pulse contribution by the frequency of the first harmonic.

[19]
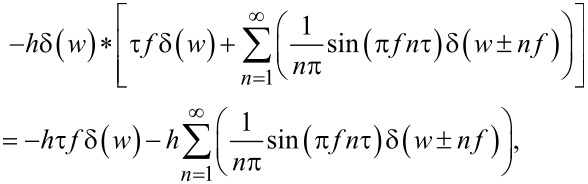


[20]
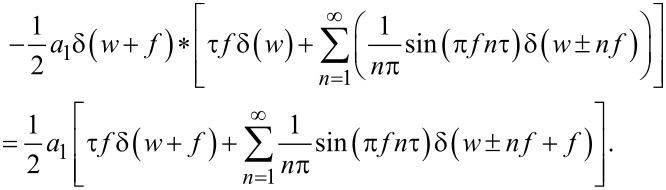


Based on the above, we conclude that the current will consist of a linear combination of shifted combs of delta functions with a sinc envelope governing the coefficients of each comb. With rapidly decreasing *a_n_* values, higher-frequency harmonics will become negligible quite rapidly. However, unlike in the noncontact (tunnelling) case, the *a_n_* values are directly involved in the current expression, without being “processed” inside of a modified Bessel function (in this case they are coefficients of delta functions). Therefore, the contribution of the higher harmonics is expected to be more significant in this case.

One can see from Equation 18 that the effective interaction time between the tip and the sample, τ, has a significant effect on the current profile. The consideration of the role of τ in Equation 18 leads to the conclusion that increasing τ increases the current magnitude and narrows the sinc envelope. Narrowing of the sinc envelope suggests more quickly decaying harmonics. This is reasonable, since increases in the contact time should lead to smoother variations in the current, which would reduce the need for very high frequency components in its Fourier transform. More quickly decaying harmonics suggest that it would be more difficult to measure a large number of them accurately, but due to the above reasons, fewer harmonics should be necessary for a proper reconstruction of the current. One can also conclude that knowledge of the tip–sample contact time would be useful in the characterization of the current profile, while, similarly, knowledge of the current as a function of time would aid in understanding the effective tip–sample contact time, which may also provide useful information regarding the mechanics of the interaction [44].

## Results

**Figure 5:**
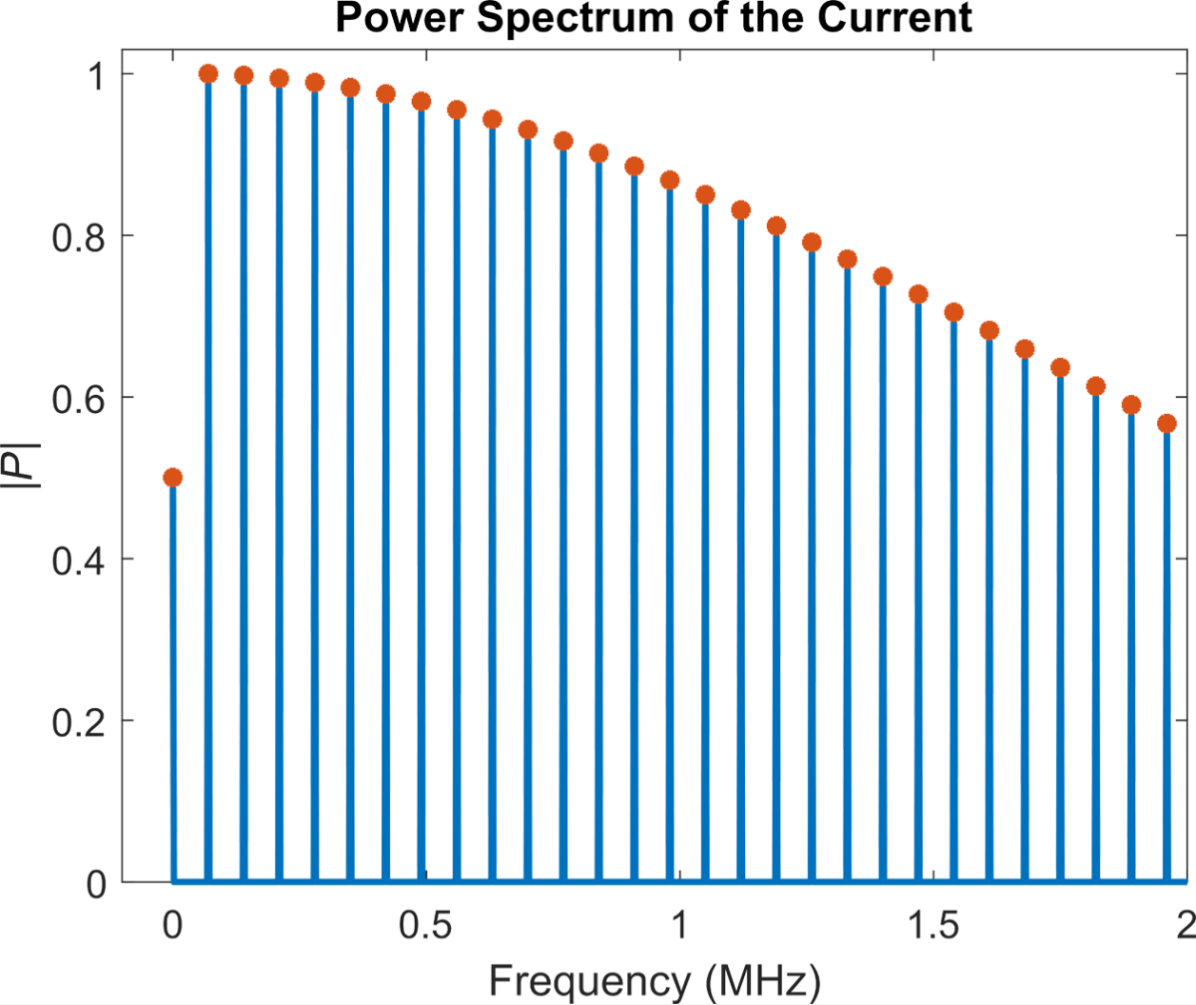
Normalised power spectrum of the current obtained for the noncontact, ideal-trajectory case. The blue lines indicate the power spectrum obtained via Fourier transform of the current, while the orange dots correspond to the predicted peaks from Equation 8. The first 50 elements of the infinite sum in Equation 8 were used for evaluating the equation. The results are in agreement with each other. Both the power spectrum and the predicted peaks are normalized. The figure also shows that the first harmonic (70 kHz), is the strongest peak in the spectrum, although the decay of the higher harmonic values is not rapid. Our calculations show that the peaks diminish to ca. 20% of the maximum peak value at a frequency of approximately 3.2 MHz, which roughly corresponds to the 46th harmonic.

In order to visualise the analytical results derived for the first case considered, we evaluate Equation 8 for a cantilever frequency of 70 kHz and an amplitude of 100 nm, with the lowest point of the trajectory being 1 nm above the surface. A tunnelling current in the form of Equation 2 is considered, with 1000-fold decay between a distance of 0 and 1 nm from the surface. As can be seen from Figure 5, the calculated frequency peaks on the power spectrum (calculated using the derived equation) match those calculated by taking the Fourier transform of the current.

In order to demonstrate the second case, we performed a numerical simulation for a dynamic AFM experiment that operates in the attractive tip–sample interaction regime. For this we have integrated the equation of motion of a spring–mass–dashpot model (Equation 21), customarily used to model dynamic AFM [45], where *m*_eff_ is the effective mass of the cantilever, *f*_0_ its natural frequency, *k* its stiffness and *Q* its quality factor:

[21]
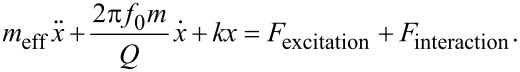


*F*_excitation_ is the sinusoidal driving force and the tip–sample interaction force, *F*_interaction_*,* is based on the Hamaker equation [42]. The simulation parameters are provided in Table 1.

**Table 1:** Simulation parameters for the spring–mass–dashpot AFM model in the attractive imaging regime (i.e., without tip–sample contact). The tip–sample interaction forces are modelled using the Hamaker equation for the case of a sphere interacting with a flat surface [42]. The imaging parameters are selected to resemble day-to-day large-amplitude experiments. The cantilever properties are similar to those of commercial cantilevers (e.g., BudgetSensors, ElectriMulti75-G conductively coated KPFM cantilevers). The Hamaker constant is chosen within the range appropriate for materials used in AFM experiments [46].

quality factor	spring constant	natural frequency	tip radius	Hamaker constant	free oscillation amplitude	resting tip distance

100	3 N/m	70 kHz	90 nm	60 × 10^−20^ J	100 nm	98 nm

**Figure 6:**
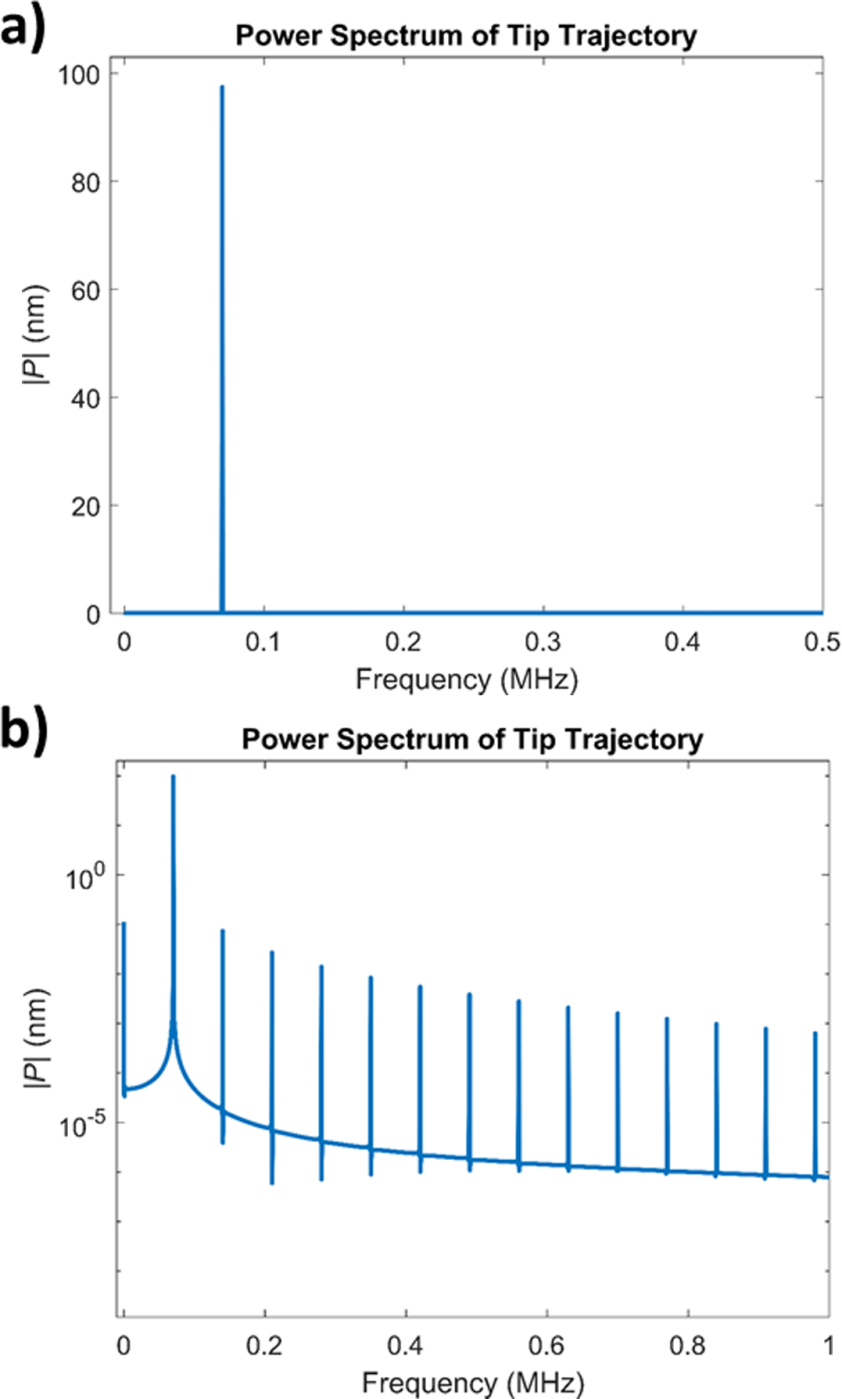
a) Power spectrum of the cantilever trajectory. The higher harmonic amplitudes are very small compared to the first harmonic amplitude, and their peaks are not visible in the spectrum with linear vertical axis. However, they are ever present and can be seen in a logarithmic plot (b), where the second harmonic is almost 1000 times smaller than the first harmonic.

**Figure 7:**
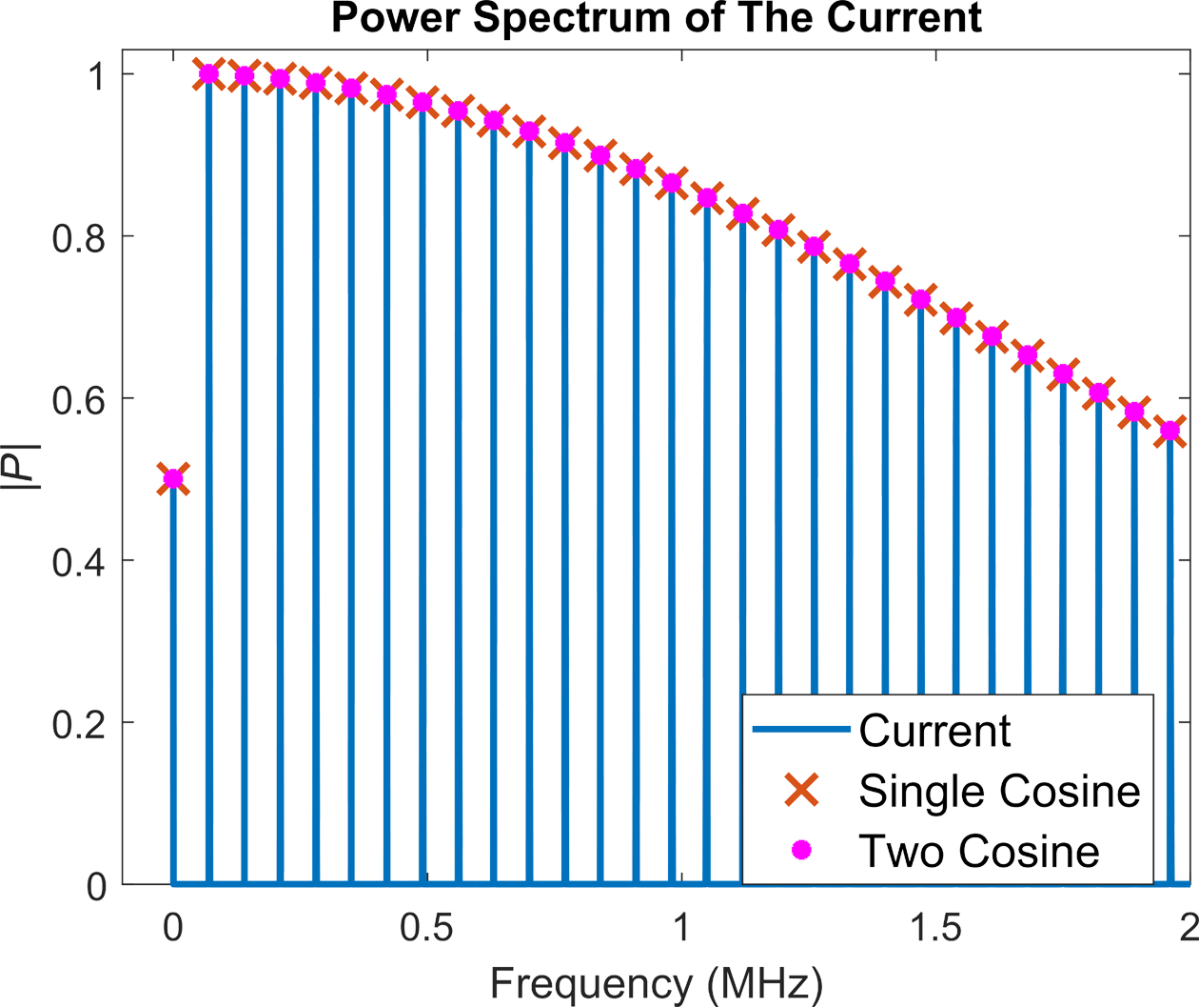
Power spectrum of the current from analytical calculations and numerical cantilever simulations for a noncontact case with attractive tip–sample forces. The blue lines correspond to the calculated power spectrum from the numerical simulation and the orange crosses correspond to the prediction from Equation 13, for the case where only one cosine term is included in Equation 12. The agreement between the two results is very good, as expected, since the higher harmonics of the tip trajectory decrease very rapidly. Pink dots are used to represent the two-cosine analytical prediction. Due to the rapid decrease of the higher harmonic amplitudes of the tip trajectory, the single- and two-cosine results fall almost on top of each other and are visually indistinguishable. The average difference between the single- and two-cosine calculations for the first 50 harmonics is 0.18%. Both calculations are normalised.

In the power spectrum of the cantilever response (Figure 6), one can observe that the *a_n_* values decrease rather rapidly, such that only the first peak contributes significantly. Therefore, the frequency representation of the tunnelling current obtained for the numerical simulation is very close to the representation based on including only one cosine term in Equation 12 (Figure 7). A calculation based on including two cosine terms in Equation 12 (that is, considering the first two harmonics of the cantilever response), also predicts the power spectrum of the current accurately, and the result is only negligibly different from the single-cosine calculation, due to the rapid decrease of the higher harmonics in the tip trajectory (also plotted in Figure 7). In contrast, the magnitude of the higher harmonics of the current does not decrease rapidly. Figure 7 shows that the magnitude of the first few harmonics is very close to the magnitude of the fundamental harmonic. This suggests that a significant number of harmonics should be detectable in an experimental setting (provided that the fundamental harmonic is detectable), and that they need to be included in order to have a valid reconstruction of the current.

**Figure 8:**
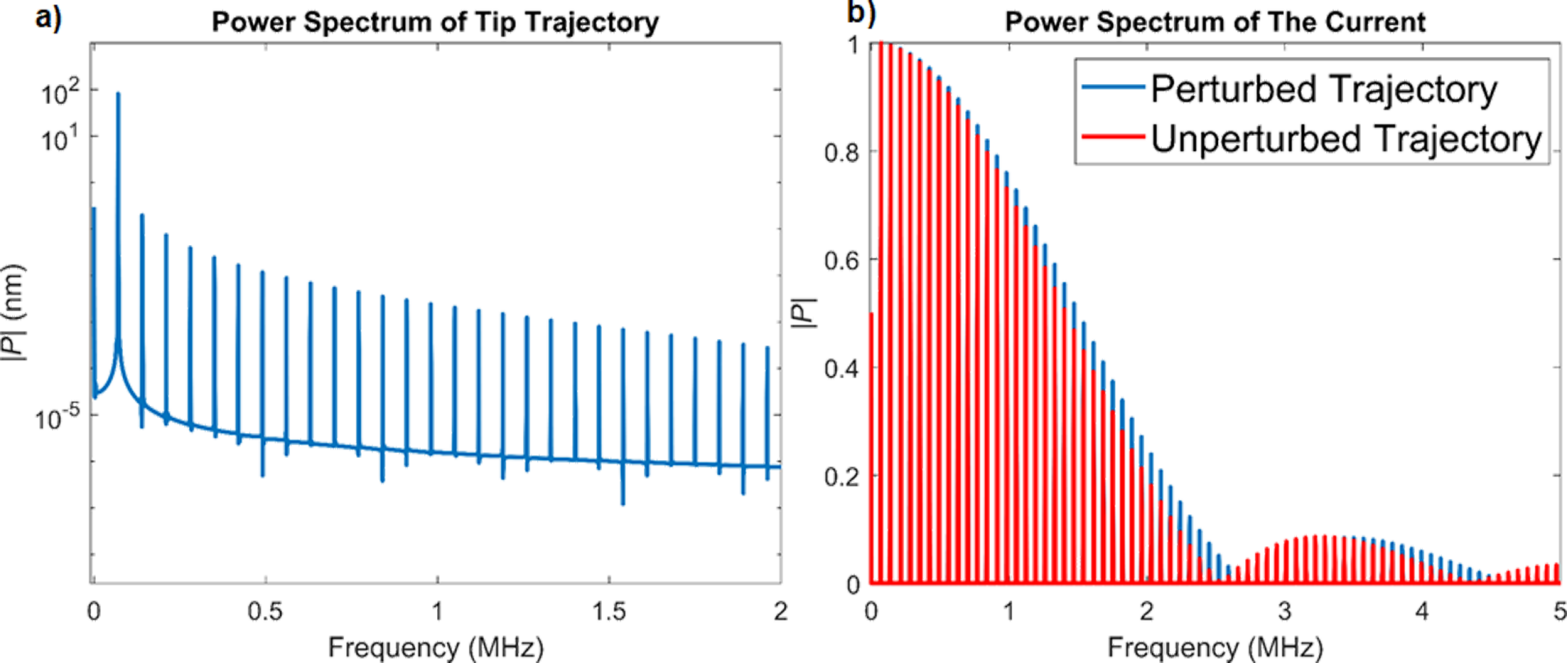
a) Power spectrum of the tip trajectory for the realistic simulation with the Hertzian repulsive interaction (the simulation parameters are provided in Table 2). The higher harmonic amplitudes of the tip oscillation are much smaller than the first harmonic amplitude but do nonetheless influence the current response. b) Comparison of the power spectrum of the current for the realistic numerical simulation with the power spectrum of the unperturbed, single-cosine trajectory. The single-cosine trajectory is designed to have the same frequency and maximum indentation as the realistic trajectory. Although the higher-harmonic amplitudes in the realistic tip trajectory are quite small compared to the first harmonic amplitude (a), the spectra of the current differ for the two trajectories considered. Both spectra exhibit the expected sinc envelope shape, with the envelope being wider when the realistic tip trajectory is considered.

The numerical simulation for case 3 is similar to that of case 2, except that only repulsive forces are considered, which are based on the Hertzian contact model (for simplicity, the attractive forces are not included). The simulation parameters can be found in Table 2. Here we first consider (i) an unperturbed cantilever trajectory, whereby the cantilever indents the surface without experiencing any tip–sample forces, and (ii) a more realistic oscillation based on the spring–mass–dashpot model of Equation 21. For a proper comparison we use the same oscillation amplitude in both cases. Figure 8 depicts the power spectrum of the realistic cantilever trajectory (Figure 8a), along with a comparison of the power spectrum of the current for the two oscillation trajectories (Figure 8b). As explained in the Introduction section, unlike the first two cases considered, here the higher harmonics of the cantilever trajectory play a more prominent role, and there are clear differences in the current calculated for the ideal and for the more realistic case. Additionally, as expected, a sinc-shaped envelope can be observed in the power spectrum of the current, whereby the amplitude of the harmonics does not follow a monotonic trend.

**Table 2:** Simulation parameters for the spring–mass–dashpot AFM model in the intermittent-contact imaging regime. For simplicity, no attractive forces are considered. The repulsive interaction is modelled using the Hertzian contact model. The simulation parameters are selected to resemble those of day-to-day intermittent contact AFM experiments. The cantilever parameters are the same as given in Table 1.

quality factor	spring constant	natural frequency	tip radius	effective elastic modulus	free oscillation amplitude	resting tip distance

100	3 N/m	70 kHz	90 nm	10 GPa	100 nm	80 nm

**Figure 9:**
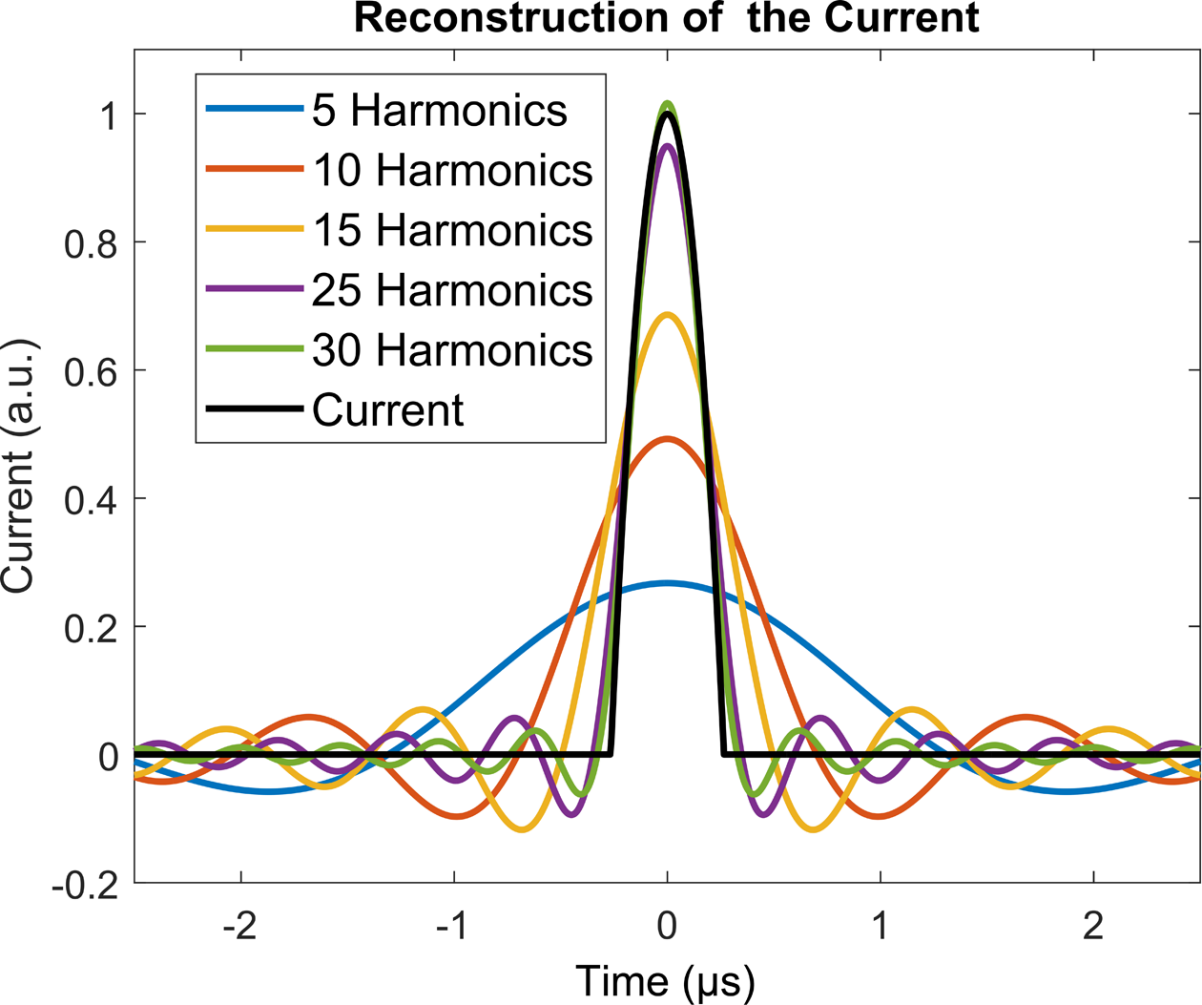
Current output obtained from the intermittent-contact simulation (black trace) and reconstruction of the current from different numbers of harmonics. As expected, inclusion of a larger number of harmonics in the reconstruction yields more accurate results. In this particular example, inclusion of 25 harmonics already leads to a very good reconstruction of the current. Since the behaviour of the harmonics coefficients as a function of frequency is not arbitrary, but rather expected to exhibit a sinc-shaped envelope, it may be possible to estimate a large number of higher harmonic amplitudes from a sparse collection of harmonics measured over a wide frequency range, such that a more accurate reconstruction is achieved.

Reconstruction of the tip–sample current should be possible if one can record its power spectrum. It is of course desirable to be able to record as many harmonics as possible, although this may not always be possible, in part due to signal-to-noise ratio limitations for very high frequencies or for frequencies near the nodes in the power spectrum (see Figure 8), and in part due to the fact that recording additional harmonics also requires additional instrumentation (i.e., lock-in amplifiers). Figure 9 illustrates the reconstruction of the current for the intermittent-contact simulation for one tip–sample impact, for different numbers of harmonics included in the reconstruction. As expected, including a larger number of harmonics leads to a current trace that is closer to the actual current. As can be seen in the figure, inclusion of 25 harmonics, whereby the 25th harmonic would still be within the range of frequencies that can be typically observed in AFM with relatively standard instrumentation (for sufficiently strong signals), already provides a very good reconstructed current (for reference, consider that the 4th eigenmode of a rectangular cantilever, which has been included in previous multifrequency AFM experiments [47], falls in the same range as the 30th harmonic). Furthermore, improvements in the reconstruction may be possible due to the fact that the shape of the power spectrum envelope may be known, as is the case for Equation 13 and Equation 18, or could be approximated from the experimental data. Specifically, during an experiment it may be possible to approximate the shape of the envelope if one has knowledge of the amplitude of a sparse collection of harmonics over a wide frequency range. With this information, one could predict the amplitude of the harmonics that have not been recorded and carry out a more accurate reconstruction of the current. It should be noted that this is possible due to the fact that all Fourier coefficients in the reconstructions discussed are real, such that if the fundamental harmonic is assigned a phase of zero, then all other harmonics should have a phase of either zero or π (see Equation 18).

## Discussion

In the previous sections we have presented a Fourier description of tunnelling and conduction current in noncontact and intermittent-contact dynamic AFM scenarios, in the context of the development of an intermittent-contact conductive AFM technique. For the noncontact case, the exponential dependence of the current with respect to the tip–sample distance led to a power spectrum for the current which contained a collection of increasing orders of modified Bessel functions of the first kind. When a repulsive interaction was considered, we again obtained a collection of harmonics in the power spectrum, although this time their coefficients were characterized by a sinc-shaped envelope, which emerges from the intermittent-contact nature of the interaction, whereby only the conductive current was considered to be significant and the tunnelling current was neglected. In all cases we obtained good agreement between the analytical expressions derived and the numerical simulations conducted, which suggests that a Fourier-based reconstruction of the current may be feasible. Nevertheless, it is important to point out anticipated challenges in the experimental implementation of the proposed method, which can be significant. We expect these challenges to arise from probe-related phenomena, data acquisition limitations, noise, the electrical nature of the sample, or some combination of all these factors.

With regards to probe challenges, it is well known that conductive tips can wear out rather easily, especially at the very apex and most especially in the case where coated tips are used, as opposed to solid conductive tips. In conventional C-AFM, small wear of the tip coating may not be as detrimental as it would be in an intermittent-contact experiment, because in the former case electrical tip–sample contact may also be established on the sides of the tip apex, particularly if indentation is significant during the experiment. However, indentation during an ICM-AFM experiment may be smaller and thus the tip contact region may be much smaller, especially under low-impact conditions. For example, in our Hertzian model simulation, the indentation is only around 0.6 nm, which limits the tip–sample contact area. In the noncontact case, the contrast is governed by the very apex of the tip, such that damage in that region may be even more problematic. Of course, the use of solid metallic tips is an alternative, although they may be more costly and the variety of parameters and geometries for which they are available is not as wide as for coated tips. In fact, coated tips can be easily fabricated starting with any non-conductive tip.

Electrical noise is anticipated to also introduce challenges. Although classical C-AFM can be affected by noise as well, the expected current magnitude in this mode of imaging should be larger than for ICM-AFM for the reasons described above, such that the current signal-to-noise ratio for the latter may be smaller. Furthermore, the current oscillations in C-AFM are of very low frequency. In contrast, ICM-AFM would involve the measurement of small currents at much higher frequencies, which would require an amplifier suitable for those conditions. Suitable instruments for experiments with relatively high conductance materials do exist (e.g., FEMTO DHPCA-100, trans-impedance amplifier [48]), which could, for example, record currents in the nanoampere regime at frequencies near 1 MHz. For experiments conducted on materials with significantly lower conductivity, different approaches need to be taken. It is possible that the use of several amplifier stages, in contrast to the use of a single trans-impedance amplifier in most conventional C-AFM setups, could improve time resolution [49]. Since the current spectrum is not expected to exhibit arbitrary frequencies, additional lock-in amplifiers can also be used. Previous researchers have provided creative examples of measurements performed on very small currents at high frequencies. For example, radio-frequency systems have been used in STM applications, such as in the work of Manassen et al. who report measurements of ca. 0.25 nA tunnelling currents at 500 MHz [50]. Such RF-STM measurements are described in detail in the work of Kemiktarak and co-workers [49]. Generally speaking, the above examples suggest that the proposed measurements could be feasible, although the equipment requirements may be considerable, since the systems would need to be replicated for each Fourier component measured simultaneously.

Creative signal processing strategies may also be necessary. For example, one possible partial solution may be the use of comb-filtering approaches similar to the implementation used by Legleiter et al. in their scanning probe acceleration microscopy (SPAM) method [51], although this would only be beneficial for harmonics that rise significantly above the noise floor, and not for those that are near the nodes of the sinc envelope. Another possible solution is the use of time averaging of the peak magnitudes at the expected harmonic frequencies, as is done in some spectroscopy procedures, for random noise to cancel itself out while physical peaks persist. Additional algorithms and smoothing could also be implemented here [52–54]. As can be seen in Figure 8b, the maximum signal intensity decreases to around 20% of the highest harmonic amplitude at close to 2 MHz in our intermittent-contact realistic example, which corresponds to the 30th harmonic. Therefore, even with a 20% noise floor, a significant number of frequency peaks may still be available for a reasonable signal reconstruction (see Figure 9). Furthermore, since the overall shape of the power spectrum is not expected to be arbitrary, it may be possible to approximate its envelope shape from a sparse collection of harmonics, as described in the Results section.

An interesting source of electrical noise, which we have observed during our initial experiments, is shaker-piezo noise. In most AFM setups, the shaker piezo is located in very close proximity to the AFM cantilever, in order to be able to perform its duties and provide the required excitation to drive the cantilever. However, a shaker piezo with a relatively high voltage amplitude (up to 10 V in our case) acts like an antenna and creates a clear peak at the oscillation frequency in the measured current. This noise is not trivial to filter out due to its location being right at the principal frequency, where we also expect our strongest current peak. This noise may also cause high-gain current amplifiers to overload and may dominate all other current signals, and this issue would be ever present regardless of the application and regardless of the type of tip used. This might be eliminated through alternate cantilever excitation methods, such as laser-based thermal excitation, although existing commercial devices with this type of excitation cannot always provide reliable and sufficiently strong excitation suitable for all types of AFM probes. This has, in fact, been the case when we have attempted to drive solid platinum probes in our laboratory, for which our thermal excitation did not impart a sufficiently large oscillation amplitude for a proper application of intermittent-contact AFM methods. Addressing this challenge may require either developments in excitation systems or probe developments. Other excitation approaches, such as magnetic excitation, may also be problematic due to the use of alternating current to drive the cantilever, which would also lead to antenna effects.

The electrical properties of the sample may play an important role in the feasibility of the proposed method. For example, in this introductory theoretical work we have treated the conductive properties of the sample as those of an Ohmic material, where the carrier response to the electric field is “immediate”. However, many materials exhibit responses that depend on the timescales of the application of the electrical interactions (e.g., on the timescale of the contact time in our case). The characterization of such materials may lead to additional challenges, where for some frequencies the carriers may not be able to respond fast enough to the intermittent interaction. Examples of such materials, which are often characterized with C-AFM, are those used in photovoltaics, which have very particular capacitive, dielectric, and impedance properties, such that the timescale of the applied bias voltage can strongly influence the result [55–56]. One additional material-related challenge, is that in some materials the measured current is already very small (this is also the case in tunnelling experiments, regardless of the material). For example, in our experiments with conductive polymers we often observe current magnitudes on the order of tens of picoamperes in C-AFM measurements. Frequency-based, amplified data acquisition systems for measurements involving a large number of harmonics have already been developed, such as for the intermodulation AFM method, which uses a battery of lock-in amplifiers [30–31], but the amplification in that case is much smaller than what would be required for ICM-AFM.

In addition to the above challenges, which may not represent an exhaustive list, there are challenges that stem from the dynamics and mechanics of an intermittent-contact operation. Besides the fact that electrical contacts would be intermittent, the nature of the contact would also be time-dependent within the contact time. This is because the indentation is constantly varying. Furthermore, the approximation of the contact area (in order to be able to make estimates of conductivity) can be very challenging for hard materials, where constant evolution of the probe geometry may occur, as well as for soft (e.g., viscoelastic [57–58]) materials, for which the indentation depends very strongly not only on the tip–sample force, but also on the rate of application of that force.

Clearly the challenges are numerous and addressing them requires significant developments and investigations, but nevertheless, we believe this is an important and potentially fruitful area of research that we have begun to explore experimentally and for which we expect to report relevant results in the near future. We also encourage related developments by other researchers.

## Conclusion

We have presented a possible method to obtain electrical property information from a sample surface using the intermittent-contact mode of AFM and Fourier analysis, considering also noncontact dynamic AFM cases. We have derived the expected current response in the frequency domain, which is in the form of harmonics of the principal frequency and discussed its shape and the significance of the various contributing terms. With the proposed models, non-measured spectral components may also be approximated. This may enable parameter-based estimation rather than model-free current reconstruction. The results suggest that reconstruction of the tip–sample current from such harmonics response is in principle feasible. However, we have also pointed out important anticipated experimental challenges that need to be addressed before realising the proposed goal.
